# Enhancing chemosensitivity of PANC1 pancreatic cancer cells to gemcitabine using ANGTPL4, Notch1 and NF-κβ1 siRNAs

**DOI:** 10.2144/fsoa-2023-0145

**Published:** 2024-05-20

**Authors:** Abdulfattah Al-Kadash, Walhan Alshaer, Ismail Sami Mahmoud, Suha Wehaibi, Malek Zihlif

**Affiliations:** 1 Department of Pharmacology, Faculty of Medicine, The University of Jordan, Amman, 11942, Jordan; 2 Cell Therapy Center, The University of Jordan, Amman, 11942, Jordan; 3 Department of Medical Laboratory Sciences, Faculty of Applied Medical Sciences, The Hashemite University, Zarqa, 13133, Jordan

**Keywords:** *ANGPTL4*, gemcitabine, *NF-κβ1*, *Notch1*, pancreatic cancer, siRNA

## Abstract

**Aim:** siRNA can silence targeted genes with lesser toxicity than therapeutic drugs. Therefore, this study aims to investigate new approaches to treat pancreatic cancer (PC) using combinations of siRNA and gemcitabine. **Methods:** Three genes, *ANGTPL4*, *Notch1* and *NF-κβ1*, were silenced using siRNA, and their anti-proliferative effects were studied in combination with gemcitabine on pancreatic cancer cell line (PANC-1) using MTT viability assay. **Results:** Our results showed a significant reduction in PANC-1 cells growth upon treating cells with gemcitabine and single and combinations of siRNA sequences specific for *ANGTPL4*, *Notch1* and *NF-κβ1* genes. **Conclusion:** Co-transfection of gemcitabine-treated PANC-1 cells with *ANGPTL4*, *Notch1* and *NF-κβ*siRNAs enhances the chemosensitivity of PANC-1 cells to gemcitabine can be a promising therapeutic approach.

## Plain language summary

Pancreatic cancer (PC) is prominent with its aggressive behavior and metastatic properties, making it one of the leading causes of cancer-related deaths worldwide. PC is associated with poor prognosis and low survival rate, with 5 years survival rate of less than 9%. Moreover, only 20% of PC patients could undergo surgery, which makes investigating new therapeutic approaches to treat PC necessary. In the current study, the chemosensitivity of pancreatic cancer cells to gemcitabine has been enhanced using a single and combination of *ANGTPL4*, *Notch1* and *NF-κβ1* siRNA.

## Abstract (plain language summary)

Pancreatic cancer is one of the aggressive cancers, ranking as the fourth most common cause of cancer-related deaths and accounting for 7.5% of all cancer-related deaths worldwide.

Recent studies illustrated the connections between gene-controlling signaling proteins like *Notch1*, *NF-κβ1* and *ANGPTL4* and pancreatic tumorigenesis, including metastasis, angiogenesis and proliferation.

Gemcitabine is the first-line chemotherapy for the treatment of pancreatic cancer.

Small interference RNA (siRNA) can be used to silence targeted genes with lesser toxicity than therapeutic drugs and has been used as a targeted therapy to enhance other chemotherapy drug effects against cancer.

The current study reveals for the first time the significant downregulation of the *Notch1* gene after silencing *the ANGPTL4* gene, which needs further investigation.

Pancreatic cancer (PC) is one of the aggressive cancers, ranking as the fourth most common cause of cancer-related deaths and accounting for 7.5% of all cancer-related deaths worldwide [1,2]. Unfortunately, the early stages of pancreatic cancer are silent. Furthermore, several risk factors have been correlated with the incidence and progression of pancreatic cancer. Some are modifiable, like smoking and high intake of alcohol, while others are non-modifiable, including male gender, ages higher than 60 years old, ethnicity and genetic variations [3,4]. The genetic variations play a fundamental role in the development of pancreatic cancer. Recent studies illustrated the connections between gene-controlling signaling proteins like *Notch1*, *NF-κβ1* and *ANGPTL4* and pancreatic tumorigenesis, including metastasis, angiogenesis and proliferation [^5-7^].

Notch1 is a transmembrane protein involved in cell differentiation, tissue proliferation, and apoptosis inside the cells. It has four types of transmembrane receptors (Notch 1–4) and five ligands (Jagged1 and Jagged 2, Delta-like 1, 3, 4) [8]. Many studies found that *Notch1* upregulation in different cancer types, including pancreatic cancer cells, was associated with lower survival rates. The complex pathways interaction between *Notch1* and *NF-κβ1*, Wnt, Ras, EGFR, and others make it a valuable therapeutic target to investigate [9].

*NF-κβ* is a transcriptional factor that has a crucial role in tumorigenesis, invasion, metastasis and chemoresistance of some drugs in PC. It consists of five members; *NF-κβ1* (p50 and p105), *NF-κβ2* (p52 and p100), RelA (p65), RelB and c-Rel. The mechanism of action of *NF-κβ* in promoting cells tumorigenesis can be justified by their increment of cyclin D1 levels, which change the cells from G1 to S phase in fibroblast. Moreover, the autocrine mechanism of *NF-κβ* allows its over-expression and activation by its activators. In addition, the antiapoptotic proteins that are produced upon *NF-κβ* activation, such as TRAF2, TRAF1 (TNF receptor Y-associated factor), Bcl (B-cell leukemia) homolog (A1/BF1-1), Bcl-xL favors mutant cell growth. These proteins can be blocked by an indirect approach by inhibiting *NF-κβ* by blocking epidermal growth factor receptors [7,10].

*ANGPLT4* is a glycoprotein and one of the VEGF family, involved in lipoprotein metabolism by inhibiting lipoprotein lipase. *ANGPTL4* can be activated by all three PPAR isoforms (PPARa, PPARb/d and PPARg), which govern its function by the isoform activated in the tissue. It is found that hypoxic conditions can enhance the expression of *ANGPTL4* by HIF-1a, leading to disturbing the junction of the epithelia to increase the permeability. Angiogenesis is a multistep process regulated by angiogenic factors like VEGF, ANG and, more recently, *ANGPTL4* [11,12].

Recently, a novel technology known as small interference RNA (siRNA) has been implemented in the gene-silencing approach and enrolled in clinical applications to enhance chemotherapeutics' effects. siRNA consists of 19–23 nucleotide double-stranded design for targeting a specific mRNA of the selected gene. The double-stranded RNA consists of a passenger (sense) and a guide (anti-sense) strand. This siRNA is activated in the cytoplasm by DICER, a member of the RNAs family. Then, the siRNA is incorporated in the RNA-induced silencing complex (RISC) and interacts with Argouate 2, which leads to the degradation of the passenger strand. The RISC active complex then attaches to the targeted mRNA by the guided strand of siRNA inside the loaded RISC complex in the cytoplasm [13].

Gemcitabine (dFdC) is a prodrug consisting of a deoxycytidine nucleoside analog that suppresses tumor proliferation by inhibiting DNA synthesis and blocking cell cycle progression in the G1/GS phase [14]. It is phosphorylated intracellularly by deoxycytidine kinase (dCK) to gemcitabine monophosphate (dFdCMP) [14,15], then phosphorylated to either gemcitabine diphosphate (dFdCDP) by pyrimidine nucleoside monophosphate kinase (NMPK) or to gemcitabine triphosphate (dFdCTP) by nucleoside diphosphate kinase (NDPK) [15]. The active metabolite dFdCTP acts as a competitive substrate of deoxycytidine triphosphate (dCTP), which cooperates at the end of the DNA strand and inhibits its elongation by the DNA polymerases [16].

In this study, we investigated the effects of silencing *NF-κB1*, *ANGPTL4* and *Notch1* genes in combination with gemcitabine on the viability of pancreatic cancer cell line (PANC-1) *in vitro*.

## Materials & methods

### siRNA sequences & primers

Table 1 shows the primer sequences used in our project, and Table 2 shows the siRNA sequences obtained from (Thermo Fisher Scientific, MA, USA).

**Table 1.** Primers sequences.

Gene	Forward (5′-3′)	Reverse (5′-3′)
*ANGPTL4*	CCTCTCCGTACCCTTCTCCA	AAACCACCAGCCTCCAGAGA
*NF-κB1*	TGGGAAGGCCTGAACAAATG	TATGGGCCATCTGTTGGCAG
*Notch1*	CTGAATTTCACTGTGGGCGG	CCCCGCAGAGGGTTGTATTG
*18s rRNA*	AGGAATTCCCAGTAAGTGCG	GCCTC ACTAAACCATCCAA

**Table 2.** siRNA sequences.

Gene	Sense	Anti-sense
*ANGPTL4*	GGUCUGGAGAAGGUGCAUAtt	UAUGCACCUUCUCCAGACCca
*NF-κB1*	GGAUUUCGUUUCCGUUAUGtt	CAUAACGGAACGAAAUCCtc
*Notch1*	GGGAGCAUGUGUAACAUCAtt	UGAUGUUACACAUGCUCCCtg
*Scrambled*	UUCUCCGAACGUGUCACGU	ACGUGACACGUUCGGAGAA

### Cell culture

Pancreatic cancer cell line (PANC-1) was obtained from the American Type Culture Collection (ATCC). Cells were cultured in DMEM medium (Caisson Labs, UT, USA), supplemented with 10% (v/v) fetal bovine serum (FBS), and 10,000 units/ml of penicillin and 10 mg/ml of streptomycin (Caisson Labs), 4.0 mM L-Glutamine (Caisson Labs). PANC-1 was incubated in a humidified incubator with 5% CO_2_ and maintained at 37 °C.

### RT-qPCR

PANC-1 cells were seeded in a six-well plate with an average seeding density of 4 × 10^5^ cells/well and incubated at 37 °C in 5% CO_2_ for 24 h to allow cell attachment. After incubation, the media was removed, and the wells were washed with 1 ml of PBS. After washing, cells were supplemented with 1 ml serum-free media and transfected with 100 nM siRNA complexed with Lipofectamine 3000 (Invitrogen, CA, USA) 1:3 molar ratio for cell transfection. Further, the plates were incubated at 37 °C in 5% CO_2_ for 6 h. After incubation, complete media is added up to 3 ml with a final FBS concentration of 10% and incubated for 72 h. Untreated cells and scrambled siRNA were used as controls for comparison.

Cells were lysed by the Trizol-hybrid method for RNA extraction using RNeasy Plus Mini Kit column (Qiagen, USA). The extracted RNA was quantified by a Nanodrop (Thermo Fisher Scientific). In order to synthesize cDNA, cDNA synthesis is required for the next step, so 0.5 μg of total RNA was reverse transcribed by GoScript™ RT Master Mix (Promega, USA) according to manufacturer protocol. The reverse transcription reaction was performed in a PCR instrument (T100™ Thermal cycler, BioRad). RT-PCR was performed as follows: 5 μl of the cDNA was mixed with 0.75 μl forward primer, 0.75 μl reverse primer, 6 μl Nuclease-free water (NFW) and 12.5 μl qPCR Master Mix. Q-PCR was performed by using a CFX96 C1000 Touch thermal cycler (Bio-Rad, CA, USA) with the following temperature settings: (i) 95 °C for 2 min, (ii) 40 cycles 95 °C for 15 s and 64 °C for 30 s. 18S rRNA was used as a reference gene. Data were analyzed using the 2^−ΔΔCT^ method.

### Detection of cell viability by MTT assay

#### IC_50_ of gemcitabine before siRNA treatment

This test is intended to measure the viability of cells in 96 wells plate by their ability to reduce the yellow tetrazolium dye (MTT); 3-(4,5-dimethylthiazol-2-yl)-2,5 diphenyltetrazolium bromide to insoluble purple-colored formazan. Around (7 × 10^3^) cells/well were seeded in a 96-well plate in 100 μl DMEM medium and incubated at 37 °C in 5% CO_2_ for 24 h in a tissue culture incubator. After incubation, the cells were treated with different concentrations of gemcitabine (0 to 2000 μM) and incubated at 37 °C in a 5% CO_2_ incubator for 72 h. Then, the MTT salt was dissolved in PBS (5 mg/ml) and 15 μl added to cells and incubated at 37 °C in a 5% CO_2_ incubator for 4 h. Later on, 50 μl of DMSO solubilization solution was added to each well and incubated for 10 min. The absorbance was measured by Glomax plate reader (Promega, WI, USA) at 570 nm.

#### siRNA transfection on MTT viability assay

PANC-1 cells were seeded in a 96-well plate with an average seeding density of 7000 cells/well and incubated at 37 °C in 5% CO_2_ for 24 h to allow cell attachment. After incubation, the media was removed, and wells were washed with 1 ml of PBS. After washing, cells were supplemented with 50 μl serum-free media and transfected with different doses of siRNA. Further, the plates were incubated at 37 °C in 5% CO_2_ for 6 h. After incubation, complete media is added up to 100 μl with a final FBS concentration of 10% and incubated for 72 h. The cell viability was detected using the same MTT assay as described above. Scrambled siRNA was used as a negative control for all siRNA formulations.

#### siRNA transfection on MTT viability assay with gemcitabine

As in the previous experiment, cells were seeded in a 96-well plate and incubated at 37 °C and 5% CO_2_. After 24 h, cells transfected with siRNA in a fixed dose (1 × 10^2^ nM), and incubated for 6 h. After that, complete media is added with a fixed dose of gemcitabine dissolved in and incubated for 72 h at 37 °C and 5% CO_2_. Then, cell viability was detected using the same MTT protocol.

### Statistical analysis

Data were entered on GraphPad Prism version 7. Two-way ANOVA was used to analyze data, and the p-value was determined to be <0.05. Values in figures represent the mean of three biological samples ± standard deviation.

## Results

### Measuring the IC_50_ of gemcitabine drug on PANC-1 cells

**Figure 1.**
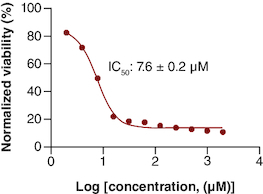
The IC_50_ of gemcitabine on PANC-1 cell line. n = 3.

The MTT viability assay of gemcitabine showed a decrease in viability as the drug concentration increased. The calculated IC_50_ of gemcitabine was 7.63 μM as shown in Figure 1. This IC_50_ is consistent with other IC_50_ values mentioned in the literature for gemcitabine in PANC-1 cell line [17].

### Gene silencing of on *Notch1*, *NF-κβ1* & *ANGPTL4*

**Figure 2.**
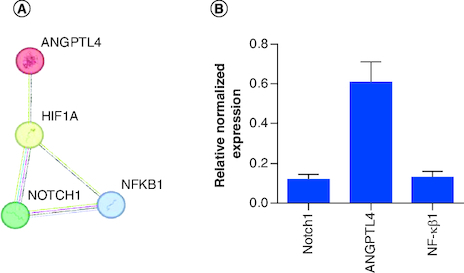
Interactions and silencing efficacy of the targeted genes. (**A)** Protein–protein interaction using STRING database, the PPI enrichment p-value: 0.0681. **(B)** Relative normalized expression of *Notch1* siRNA, *ANGPTL4* siRNA, *NF-κβ1* siRNA in cells transfected with 100 nM siRNA targeting each gene's mRNA. The results normalized to scrambled-siRNA. n = 3, values are (mean ± SD).

Different genes are overexpressed and targeted on PANC-1. The protein–protein interaction (PPI) network helped to understand the complex close shield interaction between the selected targeted genes in our study. The genes were entered into the STRING database (https://string-db.org/), and the origin was defined as ‘*Homo sapiens*’ (Figure 2A).

The expression of *Notch1*, *NF-κβ1* and *ANGPTL4*-targeted genes was investigated by RT-qPCR. Cells were transfected with 100 nM of each siRNA separately, which has an acceptable safety profile for cells. The housekeeping gene was 18s ribosomal RNA and data were analyzed using 2^−ΔΔCT^ method.

The silencing of (*Notch1, ANGPTL4 and NF-κβ1*) genes yielded an average of 88 (p < 0.0001), 39 and 87% gene silencing, respectively (Figure 2B).

### The effect of siRNA knockdown of *Notch1*, *NF-κβ1* & *ANGPTL4* genes on the viability of PANC-1 cells

**Figure 3.**
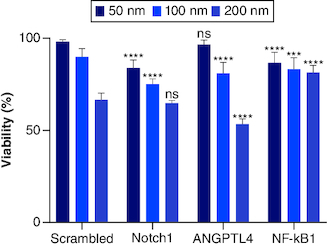
The dose-dependent assay for siRNAs (*Notch1*, *scrambled* (*SC*), *ANGPLT4* and *NF-κβ1*) from left to right, respectively. siRNAs compared with mocked transfected PANC-1 cell line. Significant differences compared with SC siRNA. ***p < 0.001; ****p < 0.0001. n = 3, values are (mean ± SD); ns: Not significant.

Dose-dependent viability assay for each siRNA (*Notch1*, *ANGPTL4* and *NF-κβ1*) has been performed on three concentrations (200, 100 and 50) nM. Each siRNA is plotted, and the values were compared with control as expressed with normalized viability of the used concentrations (Figure 3). *Notch1* gene silencing showed (64.7, 75.08 and 83.79%) viability at (200, 100 and 50) nM siRNA concentrations, respectively. Compared with scrambled (SC) siRNA - to rule out nonspecific toxicity of siRNA and off-target effects – triplicates of SC siRNA on the three different concentrations gave viability of (66.52, 89.81 and 98.06%) at (200, 100 and 50) nM siRNA concentrations, respectively. The results showed no significant differences in PANC-1 cells viability when treated with 200 nM *Notch1* siRNA compared with SC. This result can be explained by the nonspecific toxicity of the siRNA at a dose of 200 nM. While *Notch1* siRNA showed an 84% decrease in cell viability when PANC-1 cells were treated with 100 nM compared with SC. Both *ANGPTL4* and *NF-κβ1* showed dose-dependent effect with viability of (53.44, 81.14 and 96.74%) and (64.70, 75.09 and 83.80%) at (200, 100 and 50) nM siRNA concentrations, respectively.

### Single combination of *Notch1*, *NF-kβ1* & *ANGPTL4* siRNA with gemcitabine

**Figure 4.**
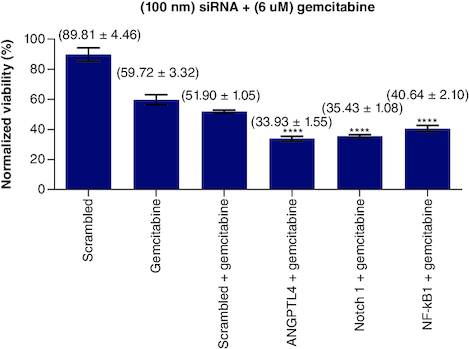
(100 nM) of each single siRNA with 6 μM gemcitabine compared with mocked transfected PANC-1 cell line. n = 3, values are (mean ± SD). Significant differences compared with SC siRNA + Gemcitabin. Statistical significance: **** indicates p < 0.0001.

To test the effect of each of siRNAs of *Notch1*, *NF-κB1* and *ANGPTL4 genes* on the sensitivity of gemcitabine, a fixed concentration of each siRNA has been added to a fixed dose of gemcitabine (6 μM) separately. The results plotted and normalized values compared with control (Figure 4). Treating PANC-1 cells with *Notch1* at (100 nM) and gemcitabine at (6 μM) showed enhanced sensitivity of PANC-1 cells to gemcitabine with 35.43% viability compared with 59.72% of gemcitabine alone and 51.90% viability of (gemcitabine and SC siRNA). *Notch1* silencing at 100 nM has 75.08% viability compared with SC and *Notch1* (1:1) molar ratio, which gives 81.69% viability (p < 0.0001). This highest effect on proliferation as compared with SC alone or SC with *Notch1* indicates the specific targeting of *Notch1*. When adding gemcitabine to our combination with siRNA, gemcitabine has a mean viability of 61.65% on 6 μM (p < 0.0001), 51.89% when combined with SC, 42.80% when combined with SC and *Notch1* (1:1) molar ratio, 35.43, 33.93 and 40.64 when combined with *Notch1*, ANGPTL4 and NF-*kβ1*siRNA, respectively (p < 0.0001).

### Single combination of siRNA (1:1) molar ratio of 100 nM with & without gemcitabine

As seen in the previous results, the addition of *Notch1* 100 nM alone with the gemcitabine has the most prominent effect compared with the nonspecific addition of SC. This supports our assumption of having a specific mechanism that integrates and manipulates the response of PANC-1 cells toward gemcitabine. However, the mechanisms of signaling pathways inside the cells are not working separately. For further investigation, we added other siRNAs to *Notch1* siRNA to study their combined effect on proliferation and sensitization of cells toward gemcitabine.

**Figure 5.**
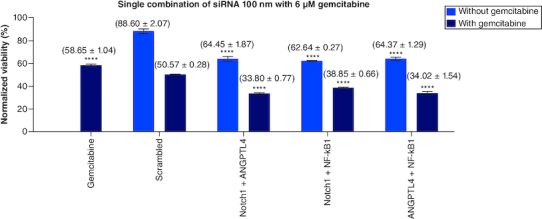
*NF-κβ1*, *Notch1, ANGPTL4* (100 nM) single combinations with 1:1 molar ratio with and without 6 μM gemcitabine compared with mocked transfected PANC-1 cell line. Significant differences compared with SC siRNA. ****p < 0.0001. n = 3, values are (mean ± SD).

*ANGPTL4* has a dose-dependent effect with an average viability of (53.44, 81.14 and 96.74%) at (200, 100 and 50 nM) concentrations, respectively. When combined with SC in a 1:1 molar ratio, we had a 72.95% viability without gemcitabine at 100 nM and 36.6% viability with (6 μM) gemcitabine compared with gemcitabine alone (58.65%). The viability of *ANGPTL4* alone with gemcitabine is 34.03%. These results show that combining gemcitabine with ANGPTL4 alone has a higher impact on PANC-1 cell proliferation than nonspecific SC (Figure 5).

## Discussion

While gemcitabine alone had 58.65% viability, the combination of *Notch1* and *ANGPTL4* siRNAs in (1:1) molar ratio with gemcitabine had the highest impact on cells' viability with 33.80% (p < 0.0001) among all studied combinations. Unfortunately, we had no significant interaction between the Notch1 and *ANGPTL4* siRNAs revealed on RT-PCR to elucidate whether this effect resulted from a cross-taking or a synergism. Accordingly, we noticed that the combination of *ANGPTL4* with Notch1 had a higher effect than *ANGPTL4* with SC (p < 0.001).

While *NF-κβ1* silencing had no significant effect on the proliferation in the dose-dependent assay alone, it decreased the viability of gemcitabine-treated cells from 59.72 to 40.64%. This enhancement further increased when combined *NF-κβ1* and *ANGPLT4* or *Notch1* (1:1) molar ratio to gemcitabine treated cells; with viability of 34.02 and 38.85%, respectively.

Upon utilizing qPCR data, the *NF-κβ1* silencing induced 40% downregulation of *Notch1* but overexpression of *ANGPTL4* by two folds. These results reveal the complex matrix of these siRNAs inside the tumor cells. Many links govern the interaction between *NF-κβ1*and *Notch1* receptor pathways; as the activation of Notch1 induces the activation of *NF-κβ1*. Moreover, *NF-κβ1* activation has strengthened the activation of the *Notch1* pathway, and once *NF-κβ1* and *Notch1* are activated, they inhibit the expression of peroxisome proliferator-activated receptor-γ (PPARγ), and therefore, they enhance tumor proliferation [18,19].

Finally, silencing *Notch1* showed a synergistic effect *with* gemcitabine, ensuring the cross mechanism that *Notch1* plays to enhance the gemcitabine sensitivity on PANC-1 cells; with decrement of the viability of gemcitabine-treated cells from 59.72% alone to a 35,43% when combined with *Notch1* In other studies, *Notch1-*activated pathways showed higher expression of drug efflux pump which contributes to higher resistance of gemcitabine on tumor cells.

We assume that targeting more than one pathway could have more effect on both the proliferation and sensitization of cells toward gemcitabine. So, we combined all the siRNA in only (1:1) molar ratio to limit the factors of the assay and compared it with and without the addition of gemcitabine. Moreover, we replaced one of the siRNA with SC each time to rule out the off-target effect and to support the coherence between these genes functions related to their downstream signals' synchronization.

As in all targeted genes, the single and combination transfected cells with *Notch1*, *ANGPTL4* and *NF-κβ1* siRNAs showed an enhanced effect of gemcitabine on the viability assays, reducing the IC_50_ of gemcitabine, which would eventually reduce its side effects and the net cost of therapy.

The combination strategy for targeting cells or multiple pathways involved in tumor progress can provide therapeutic potency, especially for undruggable cancers and decrease the chances of developing resistance to single therapy alone. The current results support that treatment of these types of cancers is multifactorial and requires targeting more than one gene on the treatment plan.

## Conclusion

The IC_50_ of gemcitabine was decreased when further transfected with *Notch1*, *NF-κβ1* and *ANGPTL4* siRNAs separately and in combinations. Moreover, the current study reveals for the first time the significant downregulation of the *Notch1* gene after silencing the *ANGPTL4* gene, which needs further investigation. This work highlights the importance of targeting multiple genes and gives new potential targets for enhancing the therapeutic effects of gemcitabine. Although this work illustrated valuable information about cells viability following the siRNA transfection and gemcitabine treatment. However, These results would be more powerful after expanding the experiments by assigning different cell proliferation assays, investigating downstream signaling pathways after combinations treatment, and developing a delivery system that can work *in vivo*.
